# RBBP6 Is Abundantly Expressed in Human Cervical Carcinoma and May Be Implicated in Its Malignant Progression

**DOI:** 10.1177/1179299X19829149

**Published:** 2019-03-11

**Authors:** Zodwa Dlamini, Thokozile Ledwaba, Rodney Hull, Sarala Naicker, Zukile Mbita

**Affiliations:** 1 Research, Innovation & Engagements Portfolio, Mangosuthu University of Technology, Durban, South Africa; 2 National Institute for Communicable Diseases, Centre for HIV and STIs, Johannesburg, South Africa; 3 Faculty of Health Sciences, Wits Medical School, Parktown, South Africa; 4 Department of Biochemistry, Microbiology and Biotechnology, University of Limpopo, Sovenga, South Africa

**Keywords:** RBBP6, Cervical cancer, apoptosis

## Abstract

RBBP6 is a novel gene encoding splicing-associated proteins. There are 3 protein isoforms (isoforms 1-3). RBBP6 isoforms 1 has been shown to interact with both p53 and Rb. It also plays a role in the induction of apoptosis and the regulation of the cell cycle. The expression of RBBP6 has been documented in several cancers but RBBP6 expression in cervical cancer has not been well studied. The aim of this study was to establish expression levels and tissue distribution of the RBBP6 gene products at both protein and messenger RNA (mRNA) levels in cervical cancer by immunocytochemistry and *in situ* hybridization (ISH). A link between RBBP6 expression, apoptosis, and cervical cancer progression was also investigated. RBBP6 mRNA was expressed in the nuclei and cytoplasm of normal and tumour cervical epithelium. In general, expression was high in the cytoplasm and nuclei of moderately differentiated and invasive carcinoma. Immunolabelling results were confirmed by image analysis and ISH experiments. Apoptosis assays using TUNEL correlated with the expression of the RBBP6 gene in all examined cases. This is the first report on the abundant expression of RBBP6 in cervical cancer and its involvement in the malignant progression of cervical cancer. Because of the high expression and corresponding pro-apoptotic activity observed in cervical cancer cells in this study, we suggest that RBBP6 is involved in the malignant progression of cervical cancer. RBBP6 proteins can therefore be targeted for therapeutic interventions against cervical cancer.

## Introduction

Cervical cancer has emerged as a leading cause of cancer-related mortalities in South African women.^1^ This can be attributed to the high incidence of human papillomavirus (HPV) infection in the sub-Saharan Africa. HPV expresses E6 and E7 oncoproteins that are known to inactivate p53 and pRB tumour suppressor proteins, respectively.^2,3^ Reactivation of p53 tumour suppressor protein through HPV E6 inactivation results in the restoration of molecular mechanisms that are meant to maintain normal homeostasis. Tumour suppressor protein, p53, has also been documented to be negatively regulated by retinoblastoma-binding protein 6 (RBBP6) and increased RBBP6 expression consequently results in tumourigenesis, whereas its knockdown results in cell growth arrest and apoptosis. This resulted in retarded growth in mice.^4^ RBBP6 is a multi-domain 250 kDa nuclear protein containing a conserved N-terminal DWNN domain.^5^ The RBBP6 proteins are translated from 2 major transcripts of 1.1 and 6.1 kb. The 1.1-kb transcript encodes a 13 kDa isoform known as isoform 3. The 6.1-kb transcript encodes 2 isoforms of 250 kDa, known as isoforms 1 and 2.^6^ RBBP6 isoform 3 consists of only the DWNN domain. This domain has a highly conserved hydrophobic region of 80 amino acids.^5^ The DWNN domain forms part of the 2 bigger isoforms: 1 and 2. In addition to the N-terminal DWNN, isoforms 1 and 2 consist of a zinc finger domain (exons 4-7), a RING finger domain (exons 8-10), a proline-rich region (exons 10-15), serine-rich (SR) and Rb-binding domains (exon 17), and a p53-binding domain (exon 18). These structural domains suggested possible roles that RBBP6 could be involved in. RBBP6 has since been implicated in a multitude of biological functions, including RNA processing,^7^ ubiquitin ligase activities,^4,8,9^ and cell cycle regulation.^6,10-13^ RBBP6 has also been implicated in several cancers,^6^ including cancers of the pancreas,^14^ lung,^15^ colon,^16^ breast,^17^ and brain.^18^ In this study, we report the abundant expression of RBBP6 in cervical cancer and its association with increasing malignant cancer progression. An important aspect of cancer research is to understand the complexities of the disease in terms of underlying principles, which, although they may be applied differentially in individual tumours, are still broadly applicable to all cancers. The ability of cancer cells to evade apoptosis is a fundamental trait and provides tumour cells with a pathway by which they may avoid drug-induced apoptosis.^19^ The increased understanding of the mechanisms of apoptosis has led to novel therapeutic approaches that may be used to stimulate tumour cells to undergo apoptosis. Therefore, the modulation of apoptosis and apoptotic regulatory factors in the development of cervical cancer are of great therapeutic interest.^20^

To determine whether RBBP6 is instrumental in cervical cancer development, transcript levels for RBBP6 were established using the reverse transcription-polymerase chain reaction (RT-PCR) and the localization of these transcripts in cervical cancer tissue sections was established using in situ hybridization. Expression levels and tissue distribution of RBBP6 gene products were established and contrasted with the expression patterns of the anti-apoptotic protein Bcl-2. Proliferation and apoptosis levels were assessed using a Ki67 antibody and TUNEL assays, respectively.

## Methods

### Sample collection

Ethical permission was obtained from the human ethics committee of the University of the Witwatersrand, Johannesburg, South Africa. Biopsy specimens of cervical carcinoma were obtained from the University of the Witwatersrand Medical School, Department of Anatomical Pathology. Tissues were then paraffin wax embedded and stored in the dark at room temperature for future studies. Sections, approximately 4-μm-thick tissue sections, were cut and adhered onto poly-l-lysine–coated slides. The sections were stained with haematoxylin-eosin to determine the histologic diagnosis of the biopsy specimens. A total of 22 (n = 22) cases of cervical cancer and 5 (n = 5) normal controls were used in this study with 10 sections per case analysed for *in situ* hybridization and immunohistochemistry. A detailed description of sample numbers per cancer description is given in Table 1.

**Table 1.** Clinicopathology of samples.

	Sample number
Normal tissue	n = 5
Well differentiated	n = 6
Moderately differentiated	n = 8
Poorly differentiated	n = 8
Total cases	n = 27

### Immunohistochemistry

Paraffin-embedded sections were dewaxed and boiled at 80°C in 0.1M sodium citrate pH 6.0 for antigen retrieval. Endogenous peroxidase was inactivated by incubating with 5% H_2_O_2_ in absolute methanol for 30 minutes (Cat# 216763, Sigma-Aldrich, St Louis, Missouri, U.S.A). Sections were then placed in blocking buffer for 1 hour to prevent non-specific binding. After this, sections were probed for the presence of immunoreactive RBBP6 using specific antibodies. Anti-human RBBP6 antibody was raised against the recombinant DWNN (RBBP6 isoform 3) domain in rabbits. The antibody was a gift from the University of the Western Cape, South Africa. Following overnight incubation at 4°C, sections were thoroughly washed and incubated with biotinylated secondary antibody conjugated with horseradish peroxidase (Dako LSAB kit peroxidase; Dako Corporation, Carpentaria, CA, USA) for 15 minutes at room temperature. The enzyme–substrate reaction was detected by the chromogen substrate, diaminobenzidine (DAB). After immunostaining, tissue sections were lightly counterstained with Mayer’s haematoxylin, dehydrated, mounted in Entellan (Merck, Darmstadt, Germany) and viewed under a light microscope. Two negative controls were used: first, the RBBP6 primary antibody was replaced by 1% bovine serum albumin in phosphate-buffered saline (PBS), and second, pre-absorbed antibody was used. Human testis tissue sections were used as the positive controls for RBBP6, as previous research in our laboratory revealed high expression levels of RBBP6 in this tissue.

### In situ hybridization

RNA probes specific to the genes encoding three RBBP6 messenger RNA (mRNA) transcripts were amplified using PCR and cloned into pGEM-T easy vectors. The cloned products were sequenced and screened against the GenBank database (National Center for Biotechnology Information [NCBI]) to verify the identity and orientation of the DWNN fragment within the vector. Antisense and sense probes were then generated using T7 or Sp6 polymerase (Roche Diagnostics, Mannheim, Germany). Paraffin wax blocks of tissue from normal and cervical cancer samples were sectioned (4 μm), dewaxed, rehydrated, and permeabilized using 20 μg/μL of proteinase K (Promega, Madison, WI, USA) for in situ hybridization. Hybridization was performed with digoxigenin (DIG)-labelled cRNA probes overnight at 55° C. After hybridization, sections were washed in 2× saline sodium citrate (SSC) and 1× SSC at 55° C and then in 0.5× SSC and 0.1× SSC at room temperature. Sections were blocked in 10% (w/v) blocking reagent (Roche Diagnostics), and then incubated for 1 hour with anti-DIG alkaline phosphatase–conjugated antibody (Roche Diagnostics) for 1 hour at room temperature. For colorimetric detection, localization was visualized with 5-bromo-4-chloro-indolyl phosphate/nitroblue tetrazolium (Roche Diagnostics, Indianapolis, IN, USA), counterstained with Mayer’s haematoxylin (Sigma). Slides were then mounted with aqueous mounting medium (Serotec, Kidlington, UK). Fluorescence detection was performed using DIG conjugated to fluorescein isothiocyanate (Roche Diagnostics). The slides were then mounted with SlowFade Light Antifade (Molecular Probes; Eugene, Oregon, USA) and viewed with a Zeiss fluorescence microscope using a 490-nm wavelength filter.

### Detection of apoptosis in tissues

Estimation of apoptosis in the tissue was determined using TUNEL assay, colorimetric TUNEL kit (Promega). Sections were dewaxed in xylene, hydrated through decreasing grades of ethanol followed by quenching of endogenous peroxidase with methanol. They were then washed in 0.85% sodium chloride. Tissues were permeabilized using 20 μg/μL of proteinase K (Promega) or 0.2% Triton X-100. The sections were fixed in 4% paraformaldehyde for 15 minutes, washed in PBS, and equilibrated for 10 minutes using equilibration buffer. The sections were then covered with a labelling mix containing biotinylated nucleotides and the enzyme, in a humidity chamber for 60 minutes at 37°C. To terminate the reaction, the sections were immersed in 2× SSC. Sections were incubated in 0.3% hydrogen peroxide and washed in PBS. They were incubated with streptavidin horseradish peroxidase diluted 1:500 in PBS and washed in PBS. The substrate reaction was developed using DAB. They were then mounted with xylene-based mounting media and viewed under a light microscope.

### Quantitative real-time PCR

Total RNA was extracted using High Pure RNA Isolation Kit (Roche Diagnostics). Reverse transcription was performed using complementary DNA (cDNA) synthesis kit for RT-PCR (Roche Diagnostics) following the manufacturer’s protocol. For quantification, LightCycler PCR was performed using the FastStart DNA master SYBR Green I kit (Roche Diagnostics). The reaction mixture consisted of 0.5 mM MgCl_2_, 0.027 μM of each PCR primer, and 1× LightCycler FastStart DNA Master SYBR Green I. Complementary DNA (2 µL) from the reverse transcription step was then added. The PCR-grade H_2_O was added in place of template to serve as a negative control. GAPDH was used as a reference gene.

The cycling conditions were as follows:

**Table:** 

Activation (1 cycle)	95°C	10 seconds
Amplification (35 cycles)	95°C	10 seconds
	50°C	10 seconds
	72°C	20 seconds
Melting curve analysis (1 cycle)	95°C	0 seconds
	65°C	30 seconds
	95°C	0 seconds
Cooling (1 cycle)	40°C	10 seconds

### Proliferation assay

Paraffin-embedded tissue sections were dewaxed and boiled in 0.1M sodium citrate buffer (pH 6.0) at 95°C for 5 minutes to unmask antigens and then allowed to cool for 20 minutes at room temperature. Endogenous peroxidase activity was quenched with 1% hydrogen peroxide in de-ionized water. Sections were then incubated in 1.5% normal blocking serum in PBS for 1 hour. Following this, sections were incubated overnight at 4°C with anti-Ki67 antibody. The following day, sections were washed and incubated with biotin-conjugated secondary antibody (Santa Cruz Biotechnology, Dallas, TX, USA). They were then incubated with avidin biotin enzyme reagent for 30 minutes, washed, and incubated in peroxidase substrate until the desired stain intensity had developed. Sections were then dehydrated and mounted in Entellan.

### Image and statistical analysis

Stained tissues were viewed and images were captured under a Zeiss light microscope. The images were analysed using an analysis software package and calibrated with a scale bar. Pixel values of DAB-labelled cells were calculated from the pixel greyscale. The DAB-labelled cells were shown to fall within a threshold of 160 to 256. A digital image is made of pixels, each with its own value on a grey scale between 1 and 256. The area of each analysed region was represented as square micrometer. The greyscale (1-256) was divided into 8 phases, each with its own upper and lower thresholds. The phases 6 to 8 represented high immunolabelling, so those were the pixels of interest. The total number of pixels that fell within these 2 phases was calculated. To get a unit that could be used across all the images, the total number of pixels representing immunolabelling were calculated per area and expressed as pixels per square micrometer. The average per image was calculated across all available data points per image and where there were more images per category; those were grouped to get 1 representative data point. For the statistical analysis, a Tukey test was performed in conjunction with a one-way analysis of variance (ANOVA). A Kruskal-Wallis test was performed as a nonparametric alternative to the 1-way ANOVA.

## Results

### Fluorescent in situ hybridization (FISH) showed progressive loss of RBBP6 mRNAs in cervical cancers

**Figure 1.**
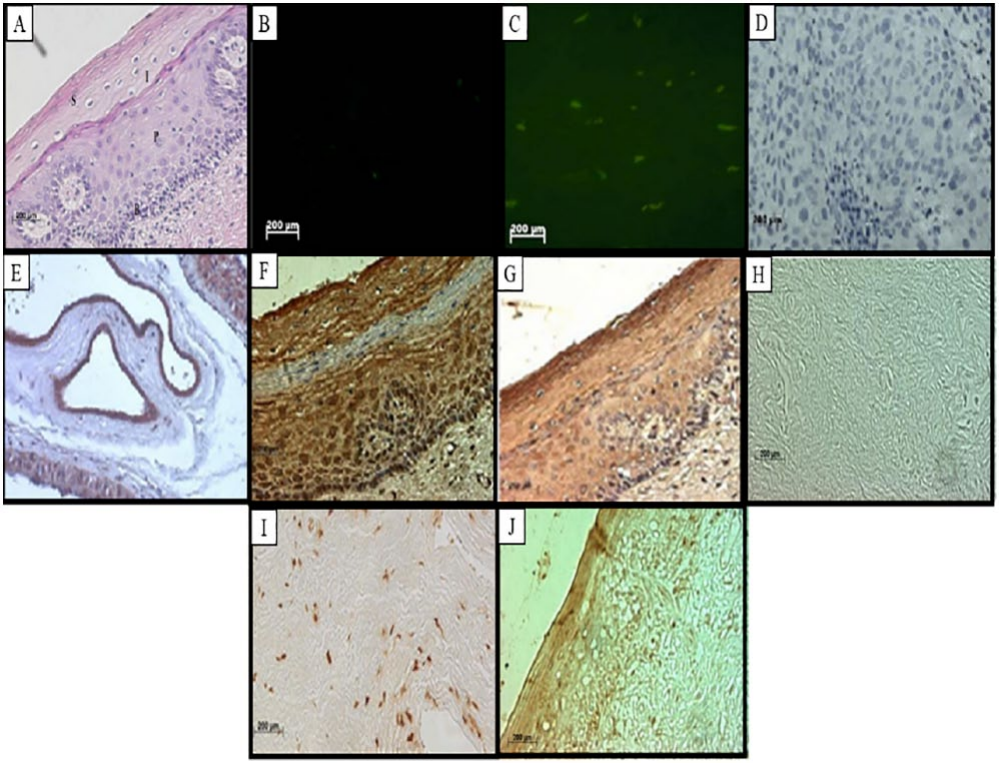
Staining of normal tissues. (A) Haematoxylin-eosin staining showing the normal epithelium that covers the ectocervix of the uterus. The different membrane layers are visible, namely, the basal (B), parabasal (P), intermediate (I), and superficial (S). (B) Fluorescent in situ hybridization showing negative staining and (C) localization of RBBP6. (D) Immunolabelling of the RBBP6 proteins in the intermediate and basal layers showed negative labelling and constituted a negative control. Localization of RBBP6 proteins in (E) the normal control (human placenta) and normal tissue section in (F) the superficial and parabasal layers. Immunohistochemistry showing the expression of Bcl-2 demonstrating specific labelling at (G) different layers of the normal epithelium, TUNEL assay results showing no labelling in a (H) negative control and intense apoptosis in the smooth muscles of a (I) normal section. Proliferation assay using Ki67 antibody shows Ki67-positive cells in the nuclei of superficial cells of (J) the basal layer. (Original magnification ×400).

**Figure 2.**
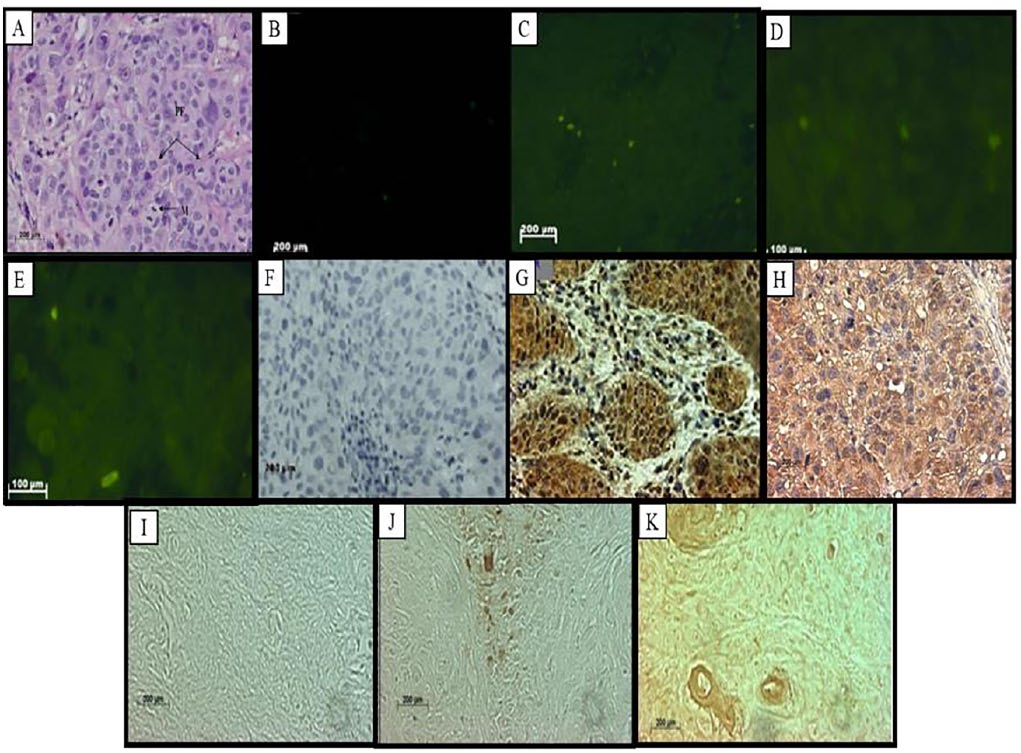
Well-differentiated squamous cell carcinoma (verrucous carcinoma). (A) Haematoxylin-eosin staining with few mitoses (M) and papillary fronds (PF). (B) Fluorescence in situ hybridization showing negative staining, (C) low levels of RBBP6 3 mRNA, (D) lower levels of RBBP6 variants 1 and 2, and (E) lower stromal expression of RBBP6 variant 1. (F) Immunolabelling of the RBBP6 proteins in the intermediate and basal layers showed negative labelling and was a negative control. (G) Immunolabelling of the RBBP6 proteins in squamous cell carcinomas (cervical carcinoma) and shows cytoplasmic accumulation. (H) Immunohistochemistry showing the expression of Bcl-2 labelling of well-differentiated squamous carcinoma. TUNEL assay results showing (I) no labelling in a negative control and (J) high apoptosis levels in the infiltrated stroma. (K) Ki67 labelling. (Original magnification ×400).

**Figure 3.**
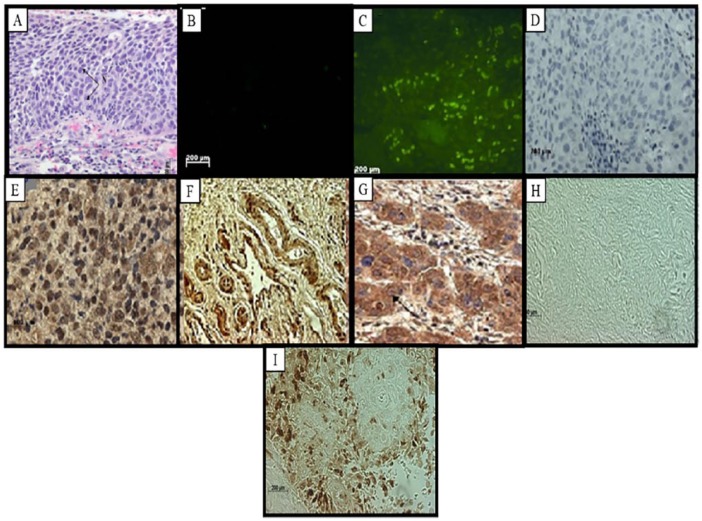
Carcinoma *in situ* showing moderate differentiation. (A) Haematoxylin-eosin staining with basophilic cytoplasm and disproportionately large nucleus (N). (B) Fluorescent in situ hybridization showing negative staining, higher expression levels of RBBP6 3 mRNA in (C) moderately differentiated carcinoma. (D) Immunolabelling of the RBBP6 proteins in the intermediate and basal layers showed negative labelling and were a negative control, whereas (E) indicates high intensity of DWNN immunostaining in the cytoplasm and the nuclei of the carcinoma cells. (F) Immunolabelling of the RBBP6 proteins in mesonephric ducts in cervical cancer. (G) Immunohistochemistry showing the expression of Bcl-2 in moderately differentiated carcinoma. (H) TUNEL assay results showing no labelling in a negative control and (I) high apoptosis levels in the infiltrated stroma. (Original magnification ×400).

**Figure 4.**
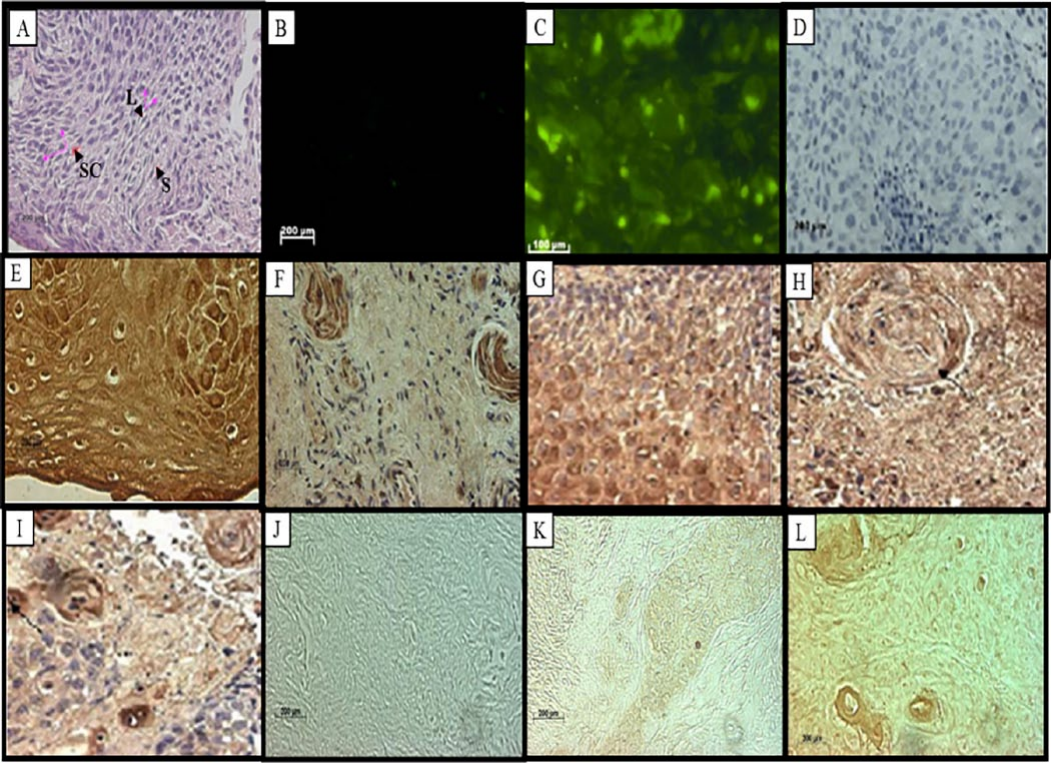
Poorly differentiated squamous cell carcinoma. (A) Haematoxylin-eosin staining showing abundant fibrovascular stroma (S), inflammatory infiltration of leucocytes (L), and irregular stratified cells (SC). (B) Fluorescent in situ hybridization showing negative staining and (C) high stromal expression of RBBP6 variant 2. (D) Immunolabelling of the RBBP6 proteins in the intermediate and basal layers showed negative labelling constituting a negative control and (E) high-intensity DWNN immunostaining in a dysplastic epithelium. (F) Strong positive DWNN immunostaining in the keratin pearls. (G and H) Immunohistochemistry showing the expression of Bcl-2 in dysplastic epithelium in cervical cancer (I) Bcl-2 labelling in keratin pearls. (J) TUNEL assay results showing no labelling in a negative control and (K) no labelling in poorly differentiated carcinoma. (L) Total proliferative status assessed by the expression of Ki67 using Ki67 antibody showing labelling of poorly differentiated carcinoma and intense labelling of the keratin pearls. (Original magnification ×400).

Using fluorescent in situ hybridization (FISH), RBBP6 variants were found to be abundantly transcribed in cervical cancer cases. Qualitatively, FISH studies showed elevated levels of the three RBBP6-mRNA transcripts in cervical cancer as compared with the normal tissues. The transcripts were localized in the nuclei of the invaded stroma, moderately differentiated islands of tumour and dysplastic epithelium (Figures 1 to 4). The FISH results showed a progressive decrease in the transcription of the RBBP6 mRNAs, as indicated in our previous work^6^; RBBP6 variant 3-mRNA transcription was lost in tumours (Figure 2C). In this article, FISH also showed that RBBP6 variants 1 and 2 are lost as cervical cancer progresses. Moderately differentiated carcinoma showed elevated levels of RBBP6 variants 1 and 2 compared with that observed in well-differentiated carcinoma (Figures 2C and 3C). In an attempt to differentiate between the two bigger transcripts, an exon 16 probe was used and there was high stromal expression of RBBP6 variant 1 (Figure 4C) compared with well-differentiated carcinoma (Figure 2D). Generally, RBBP6 mRNAs were found to be localized both in the cytoplasm and the nucleus. There were elevated levels of expression in moderately differentiated squamous cell carcinoma and the invaded stroma. But low levels of transcription were found in a normal tissue and a well-differentiated squamous cell carcinoma. The normal tissue also indicated low levels as compared with a cancerous epithelium and also showed both nuclear and cytoplasmic localization.

### RBBP6 proteins are highly expressed in cervical cancer

**Figure 5.**
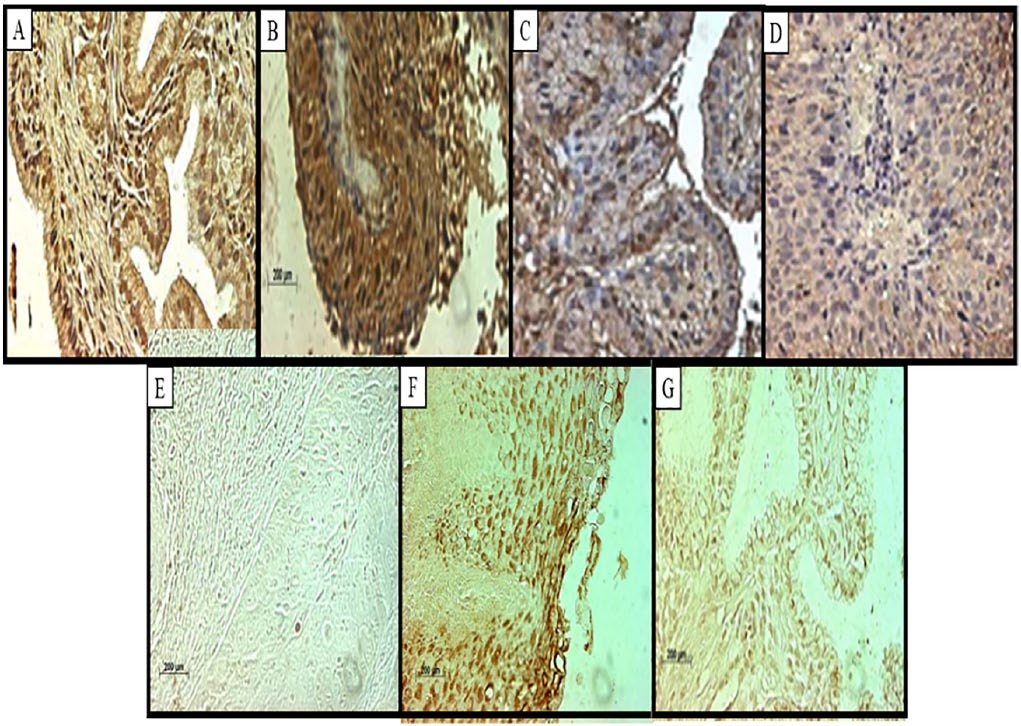
Labelling of the endocervical glands. Immunolabelling of the RBBP6 shows nuclear staining in the (A) normal endocervical glands and indicates intense staining of the (B) dysplastic endocervical glands. Immunohistochemistry showing the expression of Bcl-2 labelling in (C) normal endocervical and in (D) dysplastic endocervical glands in cervical cancer. (E) TUNEL assay results showing no labelling in the dysplastic endocervical glands. Ki67 weak staining of the nuclei of the (F) normal endocervical glands’ expression along the membranes of the (G) dysplastic epithelium. (Original magnification ×400).

**Figure 6.**
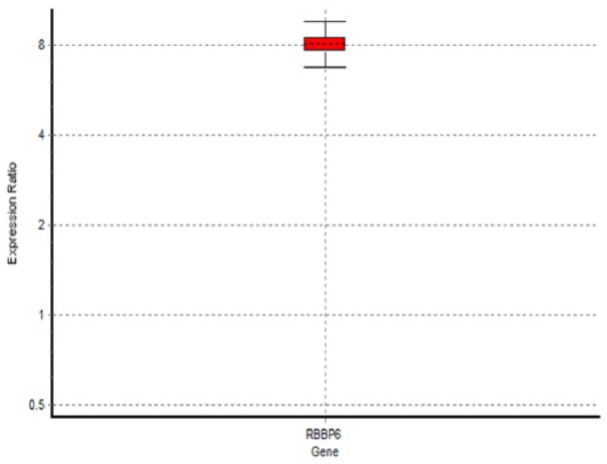
Image analysis of the localization of RBBP6 proteins in cervical tumours: Box plot showing upregulation of RBBP6 by a mean factor of 8.073 in cervical carcinoma cell lines as compared with normal cell lines.

In Figures 1 to 5, immunohistochemistry showed that RBBP6 proteins were expressed at high levels in the dysplastic epithelium, moderately and well-differentiated islands of tumours and the invaded stroma. Image analysis (Figure 6) indicated upregulation of RBBP6 by a mean factor of 8.073 (*P* = .0001) in cervical carcinoma cell lines (HeLa) as compared with a normal lung cell line (MRC-5). Immunohistochemistry showed that the RBBP6 proteins were highly expressed in moderately differentiated squamous carcinoma. Normal epithelium showed low levels of localization in the parabasal, basal, and superficial layers, but the intermediate layer showed no labelling (Figure 1E and F). Nuclear localization was observed in the basal layer, whereas the parabasal and the superficial layers showed only cytoplasmic localization. High expression levels were observed in invasive carcinoma where there was both cytoplasmic and nuclear localization. Low levels of RBBP6 proteins were observed in the stroma of the normal cervix. Poorly and moderately differentiated carcinoma showed high expression levels of RBBP6 proteins both in the cytoplasm and in the nucleus (Table 2; Figures 3G and 4E). In contrast, a well-differentiated carcinoma showed low levels of RBBP6 protein expression both in the cytoplasm and in the nucleus. Keratin pearls showed the highest levels of RBBP6 expression (Table 2; Figure 4F).

**Table 2.** Observed expression patterns of Bcl-2, Ki67 and apoptosis in relation to DWNN.

	Normal tissue	Well-differentiated carcinoma	Moderately differentiated carcinoma	Poorly differentiated carcinoma
No. of samples where DWNN is expressed, %	40/50	54/80	72/80	72/80
	80	60	90	90
No. of samples where Bcl-2 and DWNN expressed, %	32/40	20/54	32	36/72
	80	37	44	50
No. of samples where DWNN and apoptosis detected, %	20/40	33/54	36	0
	50	61	50	0
No. of samples where Ki67 proliferation marker and DWNN were expressed, %	20/40	33/54	58/72	36
	50	61	81	50

#### The relationship between RBBP6 and Bcl-2 expression is inversely proportional

Bcl-2 is a proto-oncogene often found in the inner mitochondrial membrane. It is a 24 kDa protein consisting of 239 amino acids which protects the cells from apoptosis and is localized in the long arm of the 18th chromosome. It is also found in the endoplasmic reticulum and some parts of the nuclear membrane. To give an idea of which apoptotic cascade or apoptotic components take part in the development of cervical cancer, the localization of the anti-apoptotic Bcl-2 protein was performed using immunostaining. High-intensity labelling indicates high expression of the protein and staining was mostly observed in the cytoplasm and to a lesser extent in the nucleus (Figures 1G, 2H, 3G, 4G-I, 5C and D). Immunostaining detected Bcl-2 throughout the cytoplasm with low concentrations in the normal tissue sections. The expression level was low in cervical lesions. Areas where low levels of anti-apoptotic Bcl-2 protein expression were detected coincided with areas where RBBP6 expression was high (Table 2). Upregulation of Bcl-2 was observed in dysplastic epithelium, but it was downregulated only in the intermediate and basal layers of normal epithelium and in normal endocervical glands. It was also upregulated in hyperplastic endocervical glands, islands of tumour, tunnel clusters, mesonephric ducts, and in the keratin pearls (Figure 4I). It was observed to be highly expressed in cancerous sections and downregulated in normal sections.

#### Association of apoptotic levels and RBBP6 expression in cervical cancer–linked stroma

Apoptosis detection by TUNEL revealed high apoptotic levels in the invaded stroma and moderately differentiated islands of tumours. These locations are significantly associated with RBBP6 localization (Table 2). Normal sections showed substantial levels of TUNEL-reactive nuclei in the normal stroma especially within the smooth muscle cells (Figure 1I). There was a progressive decrease in the intensity of apoptosis as histologic abnormality increased. In well-differentiated squamous carcinoma, some of the nuclei showed staining; only the surrounding stroma indicated high apoptotic levels as in Figure 2J. Poorly differentiated carcinomas showed that very few of the nuclei stained (Figure 4K), and the dysplastic endocervical glands showed a marked decrease in apoptotic bodies (Figure 5E).

### There is an indirectly proportional link between RBBP6 expression and Ki67 staining

Proliferation assays using Ki67 antibody found proliferation to be indirectly proportional to RBBP6 expression (Table 2). In Figures 1 to 5, the proliferation assay showed high levels of Ki67 expression in cancer. In normal sections, there was decreased nuclear staining in the basal layer, and no staining was observed in the parabasal and intermediate layers, but there was cytoplasmic staining in the superficial layer (Figure 1J). In dysplastic epithelium, indication of a positive reaction is present in the thickness of the epithelium. In the invasive squamous cell carcinoma, almost all cells of the surface epithelium and tumour islands in the underlying stroma were Ki67 positive (Figure 2K). Ki67-positive cells also appeared in the infiltrated stroma (Figures 3I and 4L).

## Discussion

There were increased levels of RBBP6 in cervical cancers in contrast to normal tissues suggesting that RBBP6 is involved in cervical cancer pathogenesis. RBBP6 expression significantly correlated with apoptotic levels and was indirectly proportional to Ki67 in human cervical cancers. Further characterization of this gene could lead to its use as a diagnostic marker and a potential therapeutic target for cervical cancer treatment.

RBBP6 has been implicated in mitotic apoptosis, cell cycle regulation, and carcinogenesis.^6,10,12,13^ In addition, RBBP6 has been reported to play roles in apoptosis, cell cycle, carcinogenesis, and development. Silencing of RBBP6 in mouse models resulted in a reduction in p53 polyubiquitination, which caused apoptosis and cell growth hindrance due to p53 buildup.^4^ Recently, it has been documented that accumulation of mutant p53 protein results in an increased cell proliferation rate and reduced apoptosis.^21^ RBBP6 has been shown to also contribute towards p53 accumulation, and consequently, this could lead to abrogation of apoptosis and cell growth. In this study, we showed that RBBP6 is highly expressed in tumour-associated stroma, which was directly related to apoptosis levels and indirectly associated with Bcl-2 expression. Cervical cancers also showed high proliferation rates which could be due to the inhibition of p53-dependent apoptosis. The RBBP6 mRNA was also upregulated in cervical cancer in contrast to the normal cervical tissue. The mRNA was localized both in the cytoplasm and in the nuclei. This mRNA was highly expressed in moderately differentiated squamous cell carcinoma and the surrounding stroma, but downregulated in well-differentiated squamous cell carcinoma. The probe used detected all the major splice variants. Apoptosis regulates tumour progression by counteracting cell proliferation by cell death. Therefore, upregulation of RBBP6 could link it to apoptosis regulation. Nuclear localization confirms that RBBP6 is a nuclear protein, but may function both in the cytoplasm and in the nucleus, although the biological significance of the RBBP6 protein in the nucleus remains to be established. RBBP6 has been shown to function in polyadenylation, which is crucial nuclear export. A RBBP6 protein localized within the nucleus may function to facilitate transcription or DNA replication. Alternatively, it may be performing a structural role during the progression of the cell cycle.

The RBBP6 proteins were highly expressed in the cytoplasm and some nuclei of moderately differentiated and well-differentiated carcinomas. Its high levels of cytoplasmic staining were also observed in moderate and severe dysplasia. In the normal cervix, the RBBP6 immunoreactivity was restricted to the intermediate layer, but downregulated in the other layers. The unlabelled intermediate layer suggests that oestrogen, which influences the amount of glycogen in this layer, might suppress RBBP6 expression. This is in line with the finding that overexpression of RBBP6 represses oestrogen-induced transcription.^22^ The keratinized cells also expressed this protein. This indicates that the RBBP6 proteins were expressed in high-grade lesions, which were already keratinized. The ANOVA analysis of staining showed that these differences were significant, *F* = 12; *P* = .0001. The Tukey honestly significant difference (HSD) test revealed that all pairwise differences among means were significant (*P* < 0.05). The Kruskal-Wallis test supported the ANOVA results. There were significantly elevated levels of RBBP6 protein at all grades of invasive carcinoma and those of dysplasia. The keratin pearls indicated high expression of the RBBP6 protein with a value of 35 pixels/100 µm^2^. The image analysis results correlated well with the light microscopic images. RBBP6 was upregulated in the undifferentiated cervical cancer grades in contrast to normal tissue sections. RBBP6 upregulation was directly proportional to the high apoptosis levels in cervical tumours (Figures 1 to 4). Apoptosis is thought to be the hallmark for therapeutic strategies in the treatment of cervical cancer, in which it eliminates the abnormal exponentially proliferating cells.^8^ were High levels of apoptosis were identified in poorly differentiated and moderately differentiated squamous cell carcinoma as well as the surrounding stroma. However, in well-differentiated squamous cell carcinoma, apoptosis levels were low and these are the same tissues where RBBP6 was upregulated. The significant correlation between high apoptosis levels and RBBP6 expression suggests that RBBP6 may be involved in the regulation of apoptosis. Upregulation of the p53 gene induces apoptosis, by activating the death gene *Bax* and downregulating the survival gene *bcl-2-2*. In human cervical cancer, p53 is usually mutated and it has been illustrated that p53 interacts with HPV E6 in the cytoplasm in cervical cancer.^23^ Apoptosis in HPV-infected cells is impaired by the loss of function of p53 due to its interaction with HPV E6. E6 deactivates p53 by inhibiting its phosphorylation, consequently preventing p53 cell growth inhibition.^24^

Levels of p53 were found to be elevated in severe dysplasia and invasive carcinoma.^24^ Elevated levels of p53 and Bcl-2 are linked to HPV infection and p53 inactivation by HPV E6 leads to overexpression of Bcl-2 protein in cervical cancer. In this study, we also found high levels of Bcl-2 in cervical cancer lesions where RBBP6 expression was low. This further suggests an involvement of RBBP6 in cervical cancer development. This study showed elevated levels of RBBP6 at the sites where p53 was found to be highly expressed, as reported previously.^25,26^ The high levels of RBBP6 matching the same expression pattern as p53 supports the suggestion that RBBP6-triggered apoptosis may be mediated through a p53-dependent pathway. Increased levels of apoptosis were observed with tumour cell invasion into the stroma, and this confirmed observations made in previous studies.^27^ RBBP6 may be another player in tumour cell invasion.

The concept of HPV infections indicates the decreased ability of infected cells to undergo apoptosis as a result of inactivation of the cellular tumour suppressor gene products, p53 and Rb by viral oncoproteins E6 and E7, respectively. Therefore, alterations in differentiation, proliferation and cell death were anticipated. These high levels of apoptosis were in accordance with the high expression levels of RBBP6. Indeed, Ki67 expression showed a dramatic increase in the rate of proliferation with increasing severity of dysplastic epithelium, which is in agreement with previous reports.

Ki67 showed immunoreactivity in a tumour cell population and it significantly correlates with the mitotic activity of tumour cells.^19^ The rate of proliferation increased as the tumour progressed, with an accompanied increase in cell death to maintain tissue homeostasis. High rates of proliferation suggested that cervical epithelial cells and invasive carcinoma cells entered terminal differentiation with tumour progression. The rate of proliferation was found to be indirectly proportional to RBBP6 expression. This finding indicated that Ki67 immunoreactivity provides an easily measurable index for the study of proliferating cell populations in cervical cancer. The relationship between RBBP6 and Ki67 immunoreactivity further suggests the involvement of RBBP6 in cell cycle regulation as previously reported.^6^

The overexpression of Bcl-2 proteins can inhibit apoptosis and extend cell survival; therefore, it can play an important role in carcinogenesis. A decrease in Bcl-2 expression does not necessarily indicate an increase in apoptosis because other factors regulating apoptotic processes may also be altered.^27^ The reliability of the TUNEL assay to only detect apoptotic cells has always been disputed because phagocytosed and necrotic cells can also be stained by this technique. Because of these disputes, markers of apoptosis were investigated to validate the observations concerning cell proliferation and death.

The immunocytochemical analysis of Bcl-2 indicated that its expression was restricted to the invaded stroma and downregulated in moderately differentiated carcinoma. Bcl-2 immunoreactivity was higher in mild dysplasia than in severe dysplasia or invasive carcinoma (data not shown). This suggests that apoptosis may be increased in cervical carcinoma as the anti-apoptotic protein expression level is reduced. Only limited studies have shown an apparent increase in Bcl-2 expression during the transition from mild dysplasia to invasive cancer, which implies a strong association between Bcl-2 expression and different stages of cervical cancer.^24^ As the inactivation of p53 may result from its association with viral oncoprotein E6,^28,29^ HPV infection may not only play a role in the development of squamous cell carcinoma but also might be associated with the increasing rates of proliferation due to the inhibition of p53, resulting in decreased Bax and increased Bcl-2 expression. A previous study indicated a significant correlation between the presence of HPV E6 protein and Bcl-2.^24^ This study suggests that HPV might be suppressing RBBP6 expression at the sites where Bcl-2 was highly expressed or it might be suppressing Bcl-2 at the area where RBBP6 was highly expressed.

## Conclusions

RBBP6 interacts with tumour suppressor proteins p53 and Rb, suggesting that it is involved in apoptosis. RBBP6 was highly expressed at the sites where there were high levels of apoptosis, which suggests that RBBP6 might be involved in apoptosis regulation. Bcl-2 protein was downregulated at the sites where RBBP6 was highly expressed, further supporting the notion that RBBP6 is involved in the regulation of apoptosis. Nuclear localization indicated that this gene was transcribed for a specific function, whereas the cytoplasmic staining indicated that the protein was in high demand; therefore, mRNA was translocated into the cytoplasm for the protein to be synthesized. The suspected ligase activity of RBBP6 also suggests that RBBP6 may be involved in the pathogenesis of cervical cancer and could play a vital role in the regulation of apoptosis.^8^ Therefore, this work suggests that RBBP6 is involved in apoptosis and cell proliferation during cervical cancer development.
